# Targeting CD155 by rediocide-A overcomes tumour immuno-resistance to natural killer cells

**DOI:** 10.1080/13880209.2020.1865410

**Published:** 2021-01-05

**Authors:** Wanyi Ng, Chenyuan Gong, Xuewei Yan, Guifan Si, Chen Fang, Lixin Wang, Xiaowen Zhu, Zihang Xu, Chao Yao, Shiguo Zhu

**Affiliations:** a Laboratory of Integrative Medicine, School of Basic Medical Sciences, Shanghai University of Traditional Chinese Medicine, Shanghai, PR China; b Department of Immunology and Pathogenic Biology, School of Basic Medical Sciences, Shanghai University of Traditional Chinese Medicine, Shanghai, PR China

**Keywords:** Cancer immunotherapy, high-throughput assay, immune checkpoint, natural products, non-small cell lung cancer

## Abstract

**Context:**

Therapeutic benefits of immunotherapy are restricted by cancer immune-resistance mechanisms. Rediocide-A (Red-A), a natural product extracted from Traditional Chinese Medicine, is a promising agent to battle against cancer which acts as an immune checkpoint inhibitor.

**Objective:**

To investigate the effect of Red-A on NK-cell tumouricidal activity.

**Materials and methods:**

NK cells were co-cultured with A549 or H1299 cells and treated with 10 or 100 nM Red-A for 24 h. Cells treated with 0.1% dimethyl sulphoxide (DMSO) was employed as vehicle control. NK cell-mediated cytotoxicity was detected by biophotonic cytotoxicity and impedance assay. Degranulation, granzyme B, NK cell-tumour cell conjugates and ligands profiling were detected by flow cytometry. Interferon-γ (IFN- γ) production was assessed by enzyme-linked immunosorbent assay (ELISA).

**Results:**

Red-A increased NK cell-mediated lysis of A549 cells by 3.58-fold (21.86% vs. 78.27%) and H1299 cells by 1.26-fold (59.18% vs. 74.78%), compared to vehicle control. Granzyme B level was increased by 48.01% (A549 cells) and 53.26% (H1299 cells) after 100 nM Red-A treatment. INF-γ level was increased by 3.23-fold (A549 cells) and 6.77-fold (H1299 cells) after 100 nM Red-A treatment. Red-A treatment down-regulated the expression level of CD155 by 14.41% and 11.66% in A549 cells and H1299 cells, respectively, leading to the blockade of tumour immuno-resistance to NK cells.

**Conclusions:**

Red-A overcomes immuno-resistance of NSCLCs to NK cells by down-regulating CD155 expression, which shows the possibility of developing checkpoint inhibitors targeting TIGIT/CD155 signalling to overcome immuno-resistance of cancer cells.

## Introduction

Approximately 10–15% of total lymphocytes are constituted by natural killer (NK) cells, they function as a critical role in the anti-tumour immunity (Krasnova et al. 2017). The signals to activate tumouricidal activity of NK cells are transduced when the activating receptors engaged with the activating ligands on cancer cells, it is then succeeded by killing the target cells through (a) releasing various cytokines including interferon-γ (IFN-γ) and tumour necrosis factor-alpha (TNF-α) (Wendel et al. 2008), (b) exocytosis of lytic granules such as granzymes and perforin (Voskoboinik et al. 2006) and (c) increase the expression level of some death receptor ligands, like Fas ligand (FasL) and TNF-related apoptosis inducing ligand (TRAIL) proteins (Kaplan et al. 1998).

Since NK cells recognize and eliminate target cells in a major histocompatibility complex-I (MHC-I)-unrestricted manner (Kaplan et al. 1998), NK-cell based adoptive immunotherapy has exhibited great potential in haematopoietic malignancies (Zhu et al. 2007). Also, a high number of tumour-infiltrating NK cells positively correlated with prognosis in solid tumours (Coca et al. 1997). However, tumour cells are prone to resistance to NK cell-mediated immune response due to the immunosuppressive microenvironment in solid tumours (Vitale et al. 2014). The ligands of T cell immunoreceptor with Ig and ITIM domain (TIGIT), CD155 (PVR or NECL5), are an inhibitory receptor of NK cells. CD155 is highly expressed in both human and rodent cancer cells, which is negatively correlated with prognosis. Therefore, blockade of immuno-resistant mechanisms of cancer cells represents a crucial strategy in overcoming the immunosuppressive microenvironment (Morvan and Lanier 2016).

In search of a natural substance that overcomes tumour immune resistance to NK cells, we performed a high-throughput assay to screen nearly 3000 Chinese medicine-derived small molecular compounds in our previous study (Gong et al. 2015; Xu et al. 2020). We found that rediocide-A (Red-A) extracted from the roots of *Trigonostemon rediocides* Craib (Euphorbiaceae) could potentially block tumour immune resistance to NK cells. Although it was reportedly effective against flea control (Jayasuriya et al. 2004), as antitoxins against cobra venom (Utsintong et al. 2009) or activator of conventional protein kinase C (Cui et al. 2012), without any precedence, its potential role in tumour immunotherapy had remained undiscovered. In this study, we demonstrated and shed light on how Red-A substantially overcomes tumour immuno-resistance to NK cells, suggesting that targeting CD155 is an alternative approach to promote the efficacy of NK cell-based immunotherapy.

## Materials and methods

### Cell culture

A549 and H1299 were purchased from the Cell Bank of the Chinese Academy of Sciences in China. Human peripheral blood mononuclear cells (PBMCs) were provided by the Shanghai Blood Center. For culturing A549 tumour cells, 10% of FBS (Gibco, Carlsbad, CA, 10099141) and 1% of penicillin/streptomycin (Yeasen, Shanghai, China, 60162ES76) were added to high glucose DMEM (HyClone, Logan, UT, SH30022.01). On the other hand, H1299 cells, PBMCs and expanded NK cells were cultured in RMPI 1640 (HyClone, Logan, UT, SH30809.01) supplemented with 10% of foetal bovine serum (Gibco, Carlsbad, CA, 10099141) and 1% of penicillin/streptomycin. H1299- or A549-luciferase cells were stably transfected with EGFP-fLuc-HyTk-pMGp^ac vector.

### Expansion of NK cells

For the expansion of NK cells, fresh PBMCs were incubated in RPMI-1640 cell culture medium consists of 100 U/mL of interlukin-2 (Pepro Tech, Rocky Hill, NJ, 200-02) while the frozen PBMCs were thawed before the incubation, then culturing in an incubator consisting of 5% CO_2_ at 37 °C for two weeks and irradiated feeder cells were added every week.

### Cell viability assay

A549 (5000 cells per well), H1299 (5000 cells per well) and NK cells (10,000 cells per well) were seeded into 96-well plates and treated with 0.01–100 nM of Red-A (BioBioPha, Kunming, China, BBP01506) for 24, 48 and 72 h. Cell viability of A549 or H1299 cells was evaluated by MTT (Millipore Sigma, Burlington, MA, M2128) assays while NK cells by CCK-8 (Yeasen, Shanghai, China, 40203ES60) assays using a microplate reader (BioTek Instruments, Winooski, VT, Synergy 2 Multi-Mode).

### Degranulation assay

A549, H1299 and NK cells were seeded into six-well plates in the presence or absence of 10–1000 nM of Red-A for 24 h. The pre-treated A549 or H1299 cells were cultured with NK cells at 1:1 ratio while the pre-treated NK cells were incubated in the presence or absence of A549 or H1299 cells at 1:1 ratio. Next, cells were stained with PE/Cy5-conjugated CD107α antibody (Biolegend, San Diego, CA, 555802) or isotype IgG (Biolegend, San Diego, CA, 409304) and incubated at 37 °C for 4 h, following by FITC-conjugated anti-human CD56 antibody (BD Biosciences, Franklin Lakes, NJ, 308304) or isotype IgG (BD Biosciences, Franklin Lakes, NJ, 551497) at 4 °C for 30 min, then the percentage of CD56^+^/CD107α^+^ NK cells was assessed by flow cytometry (BD Biosciences, Franklin Lakes, NJ, Accuri C6).

### Biophotonic cytotoxicity assay

A549-Luc cells (H1299-luciferase), NK cells, tumour cells or a mixture of cells in a ratio of 1:1 or 1:2 were seeded into 96-well opaque-walled plates with or without Red-A. After 24 h incubation, the substrate of luciferase (d-luciferin, PerkinElmer, Waltham, MA, 122796) was added, and luminescent intensity was read with a microplate reader. Percent lysis of target cells was calculated according to the following formula:

$$\text{lysis \%}=[1-({\mathrm{MEAN}}_{\mathrm{experiment}}-{\mathrm{MEAN}}_{\mathrm{triton}})/({\mathrm{MEAN}}_{\mathrm{medium}}-{\mathrm{MEAN}}_{\mathrm{triton}})]\times 100\%.$$

MEAN_experiment_ is the mean luminescent signal of the experimental wells, MEAN_triton_ is the background luminescent signal of target cells, while MEAN_medium_ is the mean luminescent signal of target cells being cultured alone.

### Impedance assay (Erskine et al. 2012)

xCELLigence impedance measuring station was placed in an CO_2_ container at 37 °C. Complete culture medium (50 μL) was added to the xCELLigence Eplate (Roche, Basel, Switzerland, 05469830001) to generate a background signal. A total of 5 × 10^3^ cells/100 μL of A549 cells or H1299 cells was seeded into 16 well Eplate. After 6 h, 100 μL of media in the well was removed with a pipet while 0.1% dimethyl sulphoxide (DMSO) or 100 nM Red-A with or without NK cells (5 × 10^3^) was added with a total volume of 100 μL. Impedance readings were then taken every 15 minutes for 48 h. The impedance reading which recorded as the cell index (CI) was calculated according to the following formula:

$$\text{lysis \%}=[{\mathrm{CI}}_{\mathrm{tumour}}-({\mathrm{CI}}_{\mathrm{tumour}+\mathrm{NK}})/{\mathrm{CI}}_{\mathrm{tumour}}]\times 100\%.$$

### Granzyme B detection

Red-A pre-treated A549 or H1299 cells were co-incubated with NK cells in a ratio of 1:1 for 20 min. Cells were washed and fixed using 4% paraformaldehyde at 4 °C for 40 min. Cells were then permeabilized using True-Nuclear Transcription Factor Buffer (Biolegend, San Diego, CA, 424401) after stained with anti-human CD56 (BD Biosciences, Franklin Lakes, NJ, 308304) at 4 °C for 20 min. Next, cells were stained with anti-human granzyme B (Biolegend, San Diego, CA, 515406) and intracellular granzyme B in tumour cells was detected by flow cytometry.

### NK-target conjugates

Red-A pre-treated A549 or H1299 tumour cells were co-incubated with NK cells in a ratio of 1:1 for 20 min after stained with calcein-AM (Thermo Fisher Scientific, Waltham, MA, 3099) or Cell Trace Far Red (Thermo Fisher Scientific, Waltham, MA, C34572), respectively. Cells were then fixed with 4% paraformaldehyde for 40 min. Data were assessed by flow cytometry.

### Ligand profiling assay

A549 or H1299 tumour cells were seeded into 100 mm plates at a density of 1 × 10^6^ and incubated with various concentrations of Red-A for 24 h. Next, cells were stained with fluorescence-conjugated antibodies, such as MIC-AB (Biolegend, San Diego, CA, 320908), DR4 (Biolegend, San Diego, CA, 307206), DR5 (Biolegend, San Diego, CA, 307406), CD95 (Biolegend, San Diego, CA, 305607), CD112 (BD Biosciences, Franklin Lakes, NJ, 551057), CD155 (BD Biosciences, Franklin Lakes, NJ, 337508), CD227(BD Biosciences, Franklin Lakes, NJ, 559774), HLA-ABC (Biolegend, San Diego, CA, 311410), HLA-E (Biolegend, San Diego, CA, 342606), HLA-G (Biolegend, San Diego, CA, 335910) and PD-L1 (Biolegend, San Diego, CA, 329707). After incubation of 30 min at 4 °C, the cells were washed and resuspended in TF buffer (BD Biosciences, Franklin Lakes, NJ, 554656). For indirect staining, cells were stained with primary antibodies such as ULBP1 (Santa Cruz Biotechnology, Dallas, TX, sc-53131), ULBP2 (Santa Cruz Biotechnology, Dallas, TX, sc-80419), ULBP3 (Santa Cruz Biotechnology, Dallas, TX, sc-53132), ULBP4 (Santa Cruz Biotechnology, Dallas, TX, sc-53133) and ULBP5 (Santa Cruz Biotechnology, Dallas, TX, sc-53134) at 4 °C for 30 min, followed by secondary antibodies (Biolegend, San Diego, CA, 406509405308) for 30 min at 4 °C. For each procedure, exposure to light was avoided with stained cells. Data were assessed by flow cytometry.

### Statistical analysis

All data were analysed using FlowJo 10.5.3 software (Ashland, OR), GraphPad Prism 7 (GraphPad Software, Inc., La Jolla, CA) and SPSS 21.0 statistical software (IBM Corp., Armonk, NY). Data were expressed as mean ± standard error of means (S.E.M.). Statistical comparison was assessed by *t*-test or one-way ANOVA. Multi-comparison was performed using LSD *post hoc* test. *p*< 0.05 was considered statistically significant.

## Results

### Red-A blocked immuno-resistance of NSCLCs

**Figure 1.**
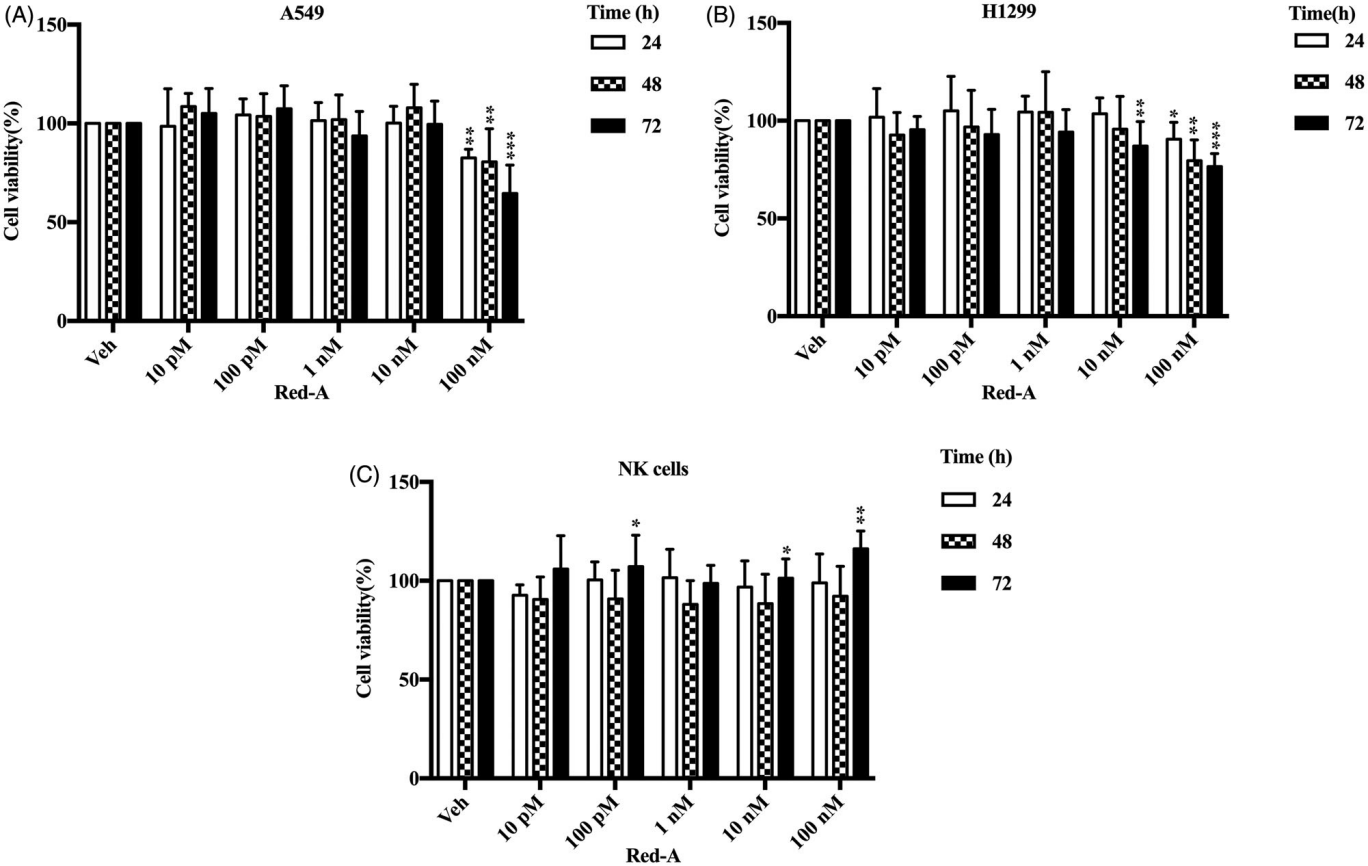
The cell viability of tumour cells and NK cells. (A) A549, (B) H1299 and (C) NK cells. Data represent mean ± S.E.M. *p< 0.05; **p< 0.01; ***p< 0.001.

**Figure 2.**
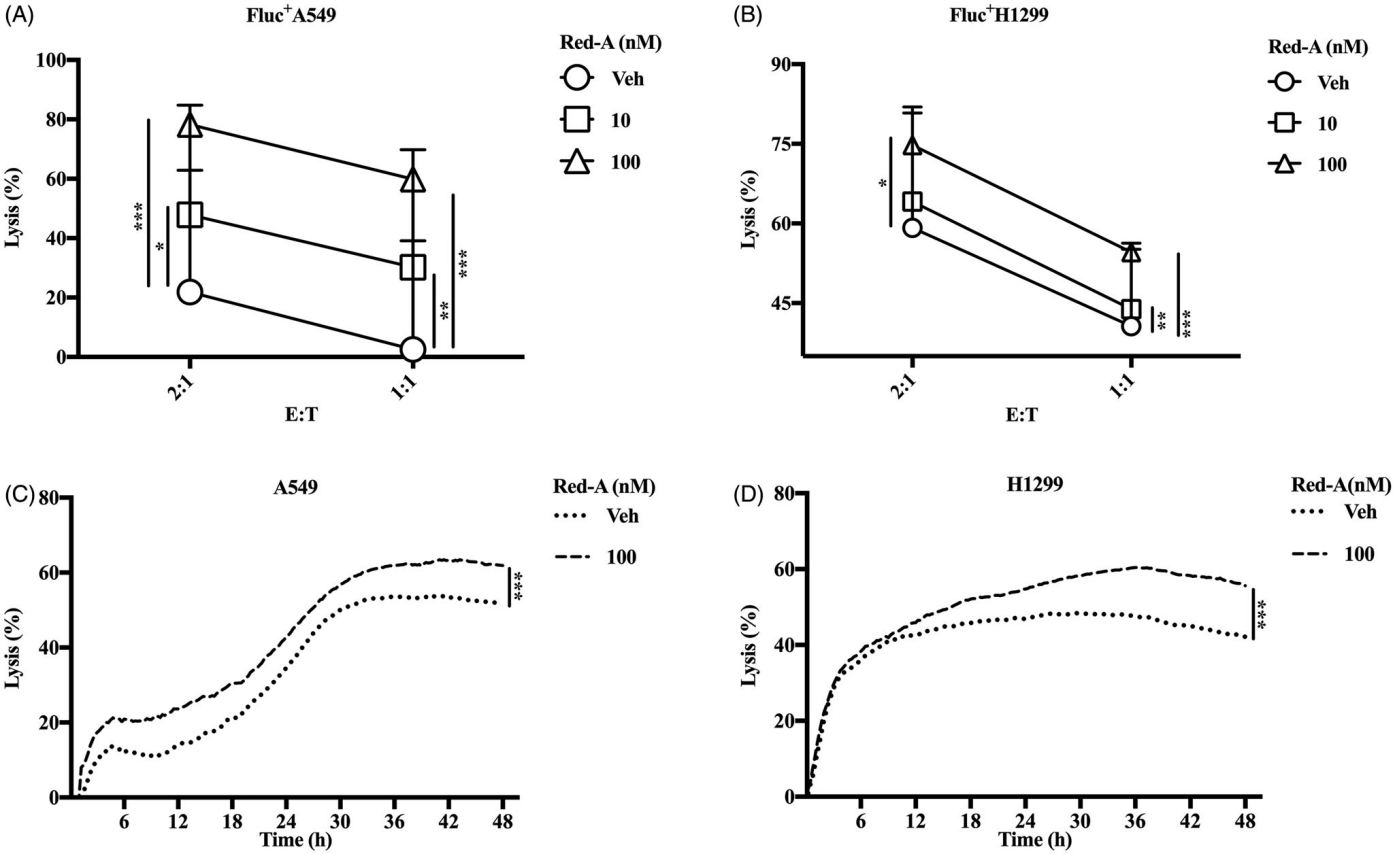
Red-A enhances NK cell-mediated killing of tumour cells. (A, B) The biophotonic cytotoxicity assay and (C, D) impedance assay were performed. E:T represents the ratio of effector cells vs. target cells. Data were pooled from three donors. Data represent mean ± S.E.M. *p< 0.05; **p< 0.01; ***p< 0.001.

Previously, we screened nearly 3000 natural products using a high-throughput assay (Gong et al. 2015; Xu et al. 2020) and found that Red-A increased NK cell-mediated killing of NSCLCs. We demonstrated Red-A could affect the viabilities of A549 and H1299 tumour cells but did not affect the viabilities of NK cells (Figure 1(A–C)). To further analyse cytotoxicity of NK cells, we investigated the effect of Red-A on the percent lysis of A549-Luc or H1299-Luc cells. Results showed that Red-A increased NK cell-mediated killing of A549-Luc and H1299-Luc cells as the concentrations increased when the ratio of effector cells against tumour cells (E:T) were 2:1 and 1:1 (Figure 2(A,B)). We used the impedance assay to detect the detachment of tumour cells following by co-culturing with NK cells. When NSCLCs adhered to electrode plates, the electrical impedance is increased, while NK cells do not bind tightly to the plates, so it does not impact the impedance much (Erskine et al. 2012). This assay further confirmed Red-A increased percent lysis of NSCLCs (Figure 2(C,D)). Taken together, these results indicated that Red-A reduced the immuno-resistance property of NSCLCs.

### Red-A increased granzyme B releasing and IFN-γ secretion by NK cells

**Figure 3.**
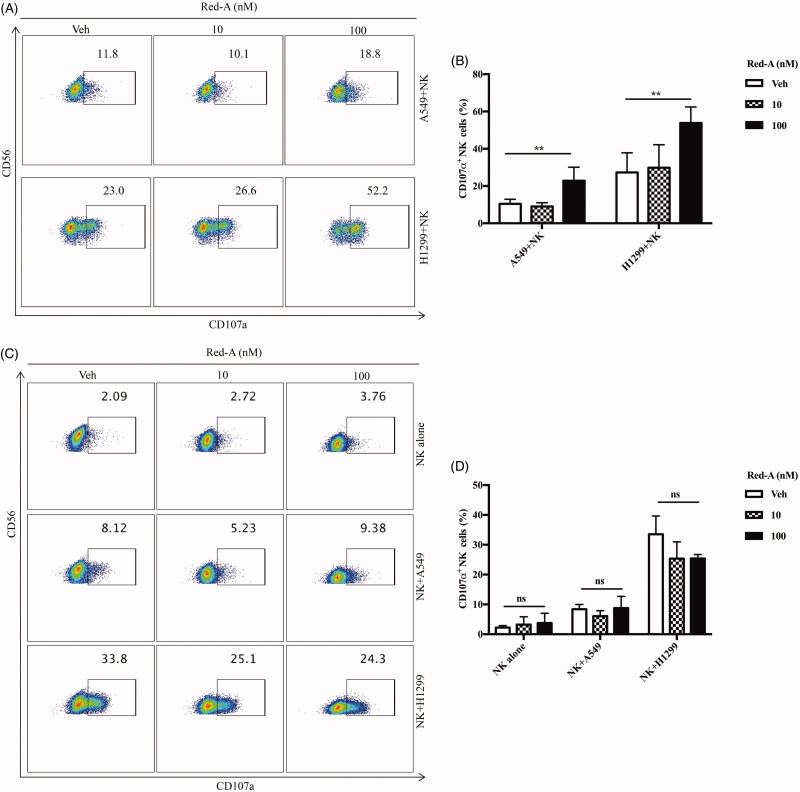
Red-A increases degranulation of NK cells. (A, B) Pre-treated A549 or H1299 cells were co-cultured with NK cells while (C, D) pre-treated NK cells were co-incubated with A549 or H1299 tumour cells. (A, C) Data represent three independent experiments while data (B, D) were pooled from three independent experiments. Data represent mean ± S.E.M. **p< 0.01. NS: non-statistical significance.

**Figure 4.**
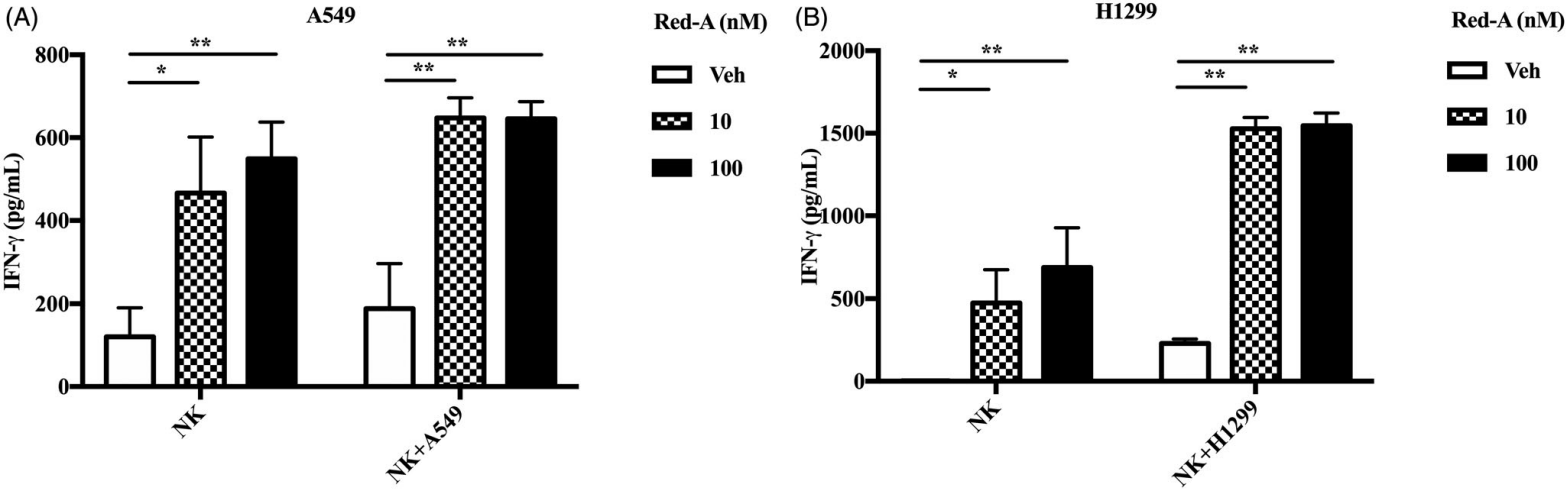
Red-A increases the secretion of IFN-γ. (A, B) NK cells were co-incubated with A549 or H1299 tumour cells in the presence or absence of Red-A, and then IFN-γ was detected by ELISA assay. Data were pooled from three independent experiments. Data represent mean ± S.E.M. *p< 0.05; **p< 0.01.

Degranulation and IFN-γ secretion are the cytotoxic factors crucial for the tumouricidal activity of NK cells. Therefore, we determined the effect of Red-A on degranulation of NK cells via detecting the percentage of CD107α positive NK cells, which reflected the degranulation level of activated NK cells (Alter et al. 2004). To investigate whether Red-A exerts a regulatory effect on NK cells or on NSCLCs, we pre-treated NK cells or NSCLCs before they were co-incubated with each other, then the percentage of CD107α positive NK cells were analysed by flow cytometry. Our results reflected a significant enhancement of CD107α positive NK cells when NSCLCs were pre-treated by Red-A (Figure 3(A,B)). However, neither the pre-treated NK cells nor the untreated NK cells co-cultured with NSCLCs showed a considerable increase in CD107α surface expression (Figure 3(C,D)). Detection of IFN-γ production also revealed that Red-A exerts its regulatory effect on NSCLCs rather than on NK cells, which leads to the increase of NK cell-mediated killing of NSCLCs (Figure 4(A,B)).

**Figure 5.**
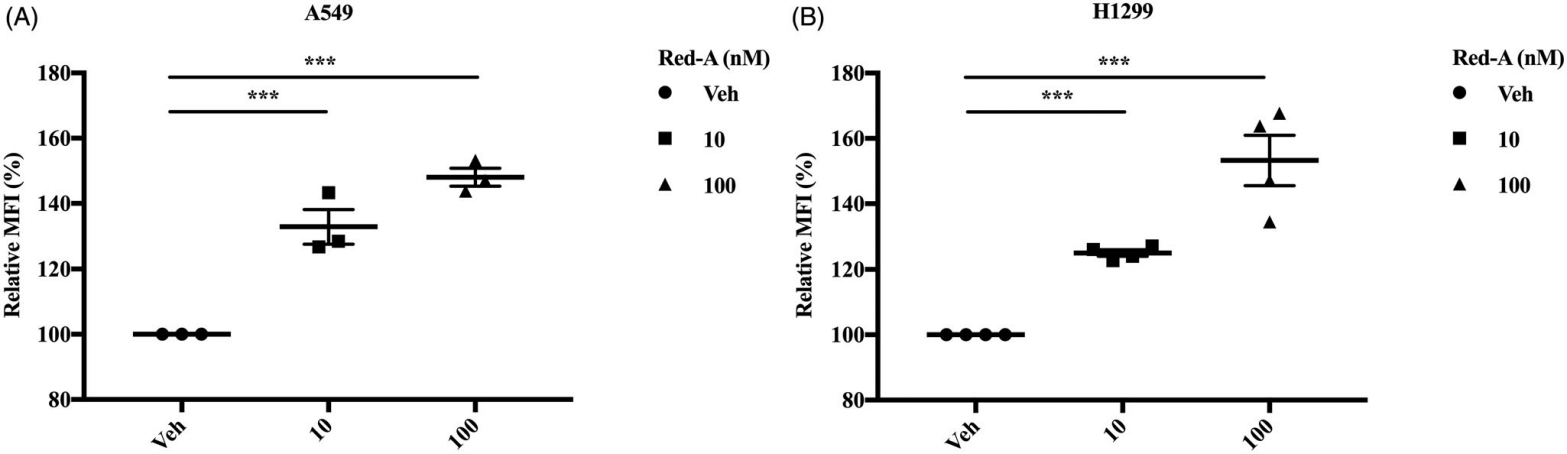
Red-A increases the releasing of granzyme B. (A, B) A549 or H1299 cells were co-cultured with NK cells in different concentrations of Red-A, then granzyme B level in A549 and H1299 tumour cells was assessed. Data A are a representative of three independent experiments while data B were pooled from three independent experiments. Data represent mean ± S.E.M. ***p< 0.001.

Furthermore, NK cells kill tumour cells by releasing cytolytic granules such as granzyme B, which induce apoptosis in tumour cells. Therefore, we accessed the granzyme B level in A549 and H1299 tumour cells derived from NK cells and found that Red-A increased NK cell-derived granzyme B in NSCLCs (Figure 5(A,B)). Overall, Red-A could enhance NK cell-mediated killing by increasing the release of lytic granules.

### Red-A down-regulates the expression of CD155

**Figure 6.**
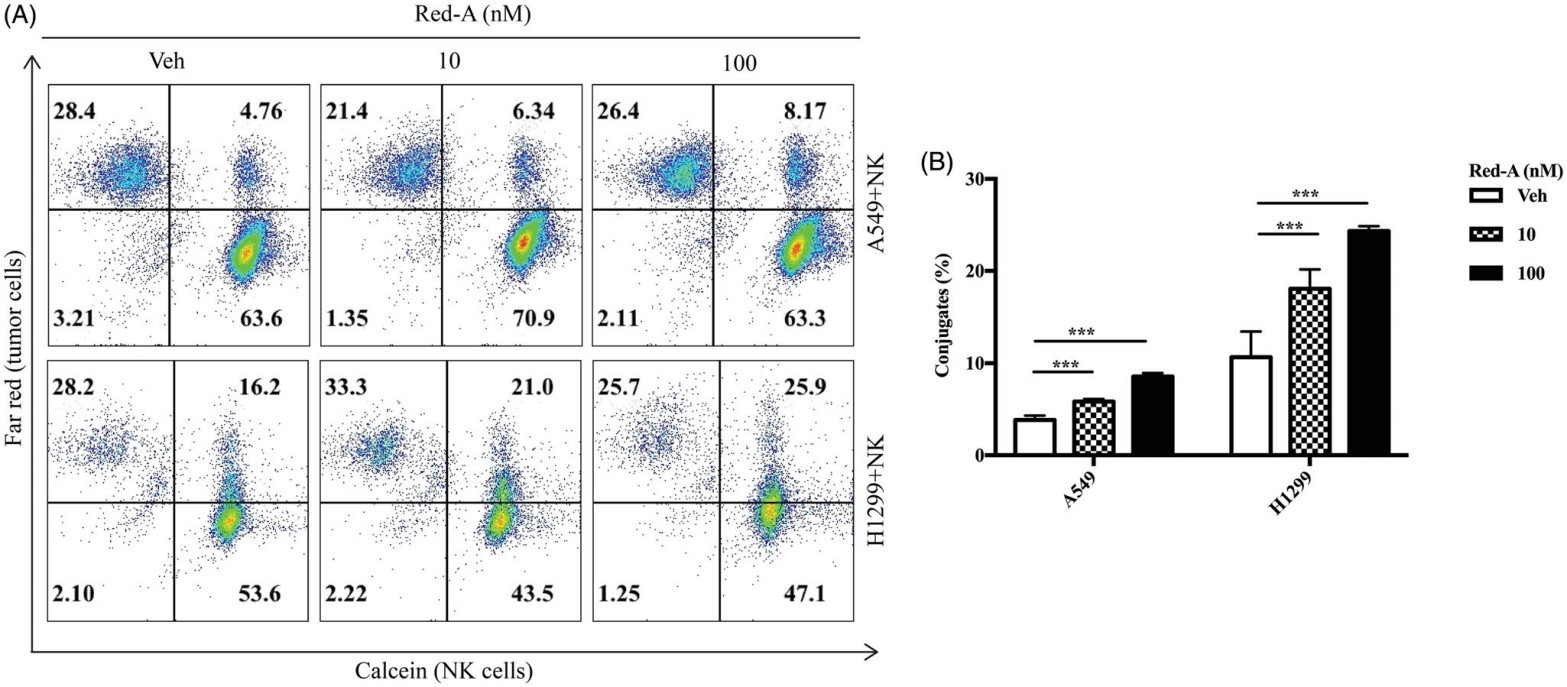
Red-A enhances the conjugation of NK cells and NSCLC cells. (A, B) The population of Far Red*/*calcein double positive cells was detected by flow cytometry to determine the conjugation of NK cells and tumour cells. Data A are a representative of three independent experiments while data B were pooled from three independent experiments. Data represent mean ± S.E.M. ***p< 0.001.

We then determined the underlying mechanism by which Red-A blocked tumour immuno-resistance to NK cells. As mentioned before, one among the three ways in which NK cells killing target cells, inducing apoptosis through FasL and TRAIL proteins. NK cells recognize target cells by engaging the receptors expressed on the surface of NK cells with the ligands expressed on the surface of tumour cells, which regulates the balance between activating and inhibitory signals (Raulet and Guerra 2009; Liu et al. 2019). We found that Red-A increased NK cell-tumour cell conjugates (Figure 6(A,B)).

**Figure 7.**
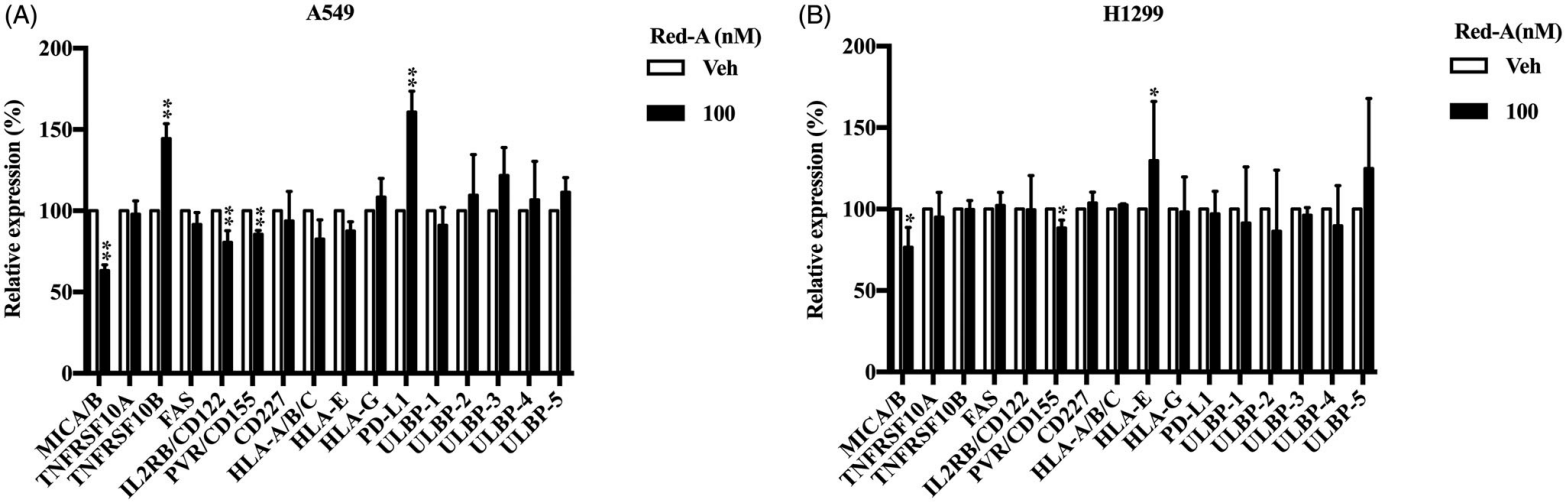
Red-A down-regulated CD155 expression level. (A, B) The activating ligands, inhibitory ligands and death receptors of NK cells were analysed by flow cytometry. Data were pooled from three independent experiments. Data represent mean ± S.E.M. *p< 0.05; **p< 0.01.

To investigate how Red-A increased recognition of NSCLCs by NK cells, we analysed the ligands expression repertoire on NSCLCs by flow cytometry. Red-A was found to decrease the expression of PVR/CD155, but it has no effect on NK cells inhibitory or activating ligands, including HLA (MHC-I)-A/B/C, HLA-E, HLA-G, MUC1 (Mucin 1, cell surface-associated)/CD227, PD-L1 (programmed death-ligand 1) for inhibitory ligands, and MIC-A/B, IL2RB/CD122, ULBP1-5 for activating ligands. Red-A did not regulate the expression of TNFRSF10A/DR4, TNFRSF10B/DR5 and FAS/CD95 in A549 (Figure 7(A)) and H1299 (Figure 7(B)) cells. A recent study demonstrated slower tumour growth and decrease in chances of metastases as displayed by CD155, to the contrary, exerted a much greater suppressive effect on tumour growth and metastasis (Li et al. 2018). Together, these results suggested that Red-A reduced inhibitory signals elicited by CD155, resulting in blockade of tumour immuno-resistance of NK cells.

## Discussion

Natural products have been a promising subject to improve NK cell therapeutic efficacy (Yao et al. 2018). Through a series of high-throughput screening assay of nearly 3000 Chinese medicine-derived natural substances, we are glad to discover Red-A and its capacity to enhance the ability of NK cells significantly. It was reported as role in flea control (Jayasuriya et al. 2004), antitoxins against cobra venom (Utsintong et al. 2009) or activator of conventional PKC (Cui et al. 2012). However, it was unknown about its role in tumour immunotherapy. Our study showed that Red-A enhanced NK cell-mediated killing by enhancing their degranulation and the production of granzyme B and IFN-γ.

Mechanistically, Red-A enhanced NK cell mediated NSCLC killing by down-regulating PVR/CD155. CD155 is a member of the nectin-like family of proteins which is reported as a therapeutic target in immunotherapy (Kucan Brlic et al. 2019). CD155 binds to DNAM-1 to trigger activating signals or binds to TIGIT and CD96 to trigger inhibitory signals (Martinet and Smyth 2015; Bowers et al. 2017). The balance between activating and inhibitory signals is often disturbed in the tumour microenvironment, which leads to the dysfunction of tumour-infiltrating NK cells. Overexpression of CD155 was shown to induce tumour immuno-resistance (Kono et al. 2008). CD155 is correlated with poor prognosis (Nishiwada et al. 2015). Data have shown that CD155 mediated both T cell and NK cell dysfunction via binding to TIGIT (Yu et al. 2009; Bi et al. 2014; Joller et al. 2014; Sanchez-Correa et al. 2019). Blockade of CD155/TIGIT signalling restored anti-tumour immunity mediated by NK cells (Zhang et al. 2018). In this study, we found that Red-A blocked tumour immuno-resistance by down-regulating CD155. Thus, targeting CD155 by natural products might offer an opportunity for checkpoint blockade therapy (Carlsten et al. 2010; Gao et al. 2017; Bronte 2018).

## Conclusions

Immune checkpoint inhibitors show the potential to cure cancer. Red-A down-regulated CD155 expression in NSCLCs and unleashed NK cells to kill tumour cells, which shows the potential therapeutic value of developing checkpoint inhibitors targeting TIGIT/CD155 signalling.
